# A Clinical-Radiomics Nomogram for Preoperative Prediction of Lymph Node Metastasis in Gallbladder Cancer

**DOI:** 10.3389/fonc.2021.633852

**Published:** 2021-09-22

**Authors:** Xingyu Liu, Xiaoyuan Liang, Lingxiang Ruan, Sheng Yan

**Affiliations:** ^1^Department of Hepatobiliary and Pancreatic Surgery, The Second Affiliated Hospital Zhejiang University School of Medicine, Hangzhou, China; ^2^Department of Radiology, The First Affiliated Hospital Zhejiang University School of Medicine, Hangzhou, China

**Keywords:** gallbladder cancer, radiomics, computed tomography, lymph node metastasis, nomogram

## Abstract

**Objectives:**

The aim of the current study was to develop and validate a nomogram based on CT radiomics features and clinical variables for predicting lymph node metastasis (LNM) in gallbladder cancer (GBC).

**Methods:**

A total of 353 GBC patients from two hospitals were enrolled in this study. A Radscore was developed using least absolute shrinkage and selection operator (LASSO) logistic model based on the radiomics features extracted from the portal venous-phase computed tomography (CT). Four prediction models were constructed based on the training cohort and were validated using internal and external validation cohorts. The most effective model was then selected to build a nomogram.

**Results:**

The clinical-radiomics nomogram, which comprised Radscore and three clinical variables, showed the best diagnostic efficiency in the training cohort (AUC = 0.851), internal validation cohort (AUC = 0.819), and external validation cohort (AUC = 0.824). Calibration curves showed good discrimination ability of the nomogram using the validation cohorts. Decision curve analysis (DCA) showed that the nomogram had a high clinical utility.

**Conclusion:**

The findings showed that the clinical-radiomics nomogram based on radiomics features and clinical parameters is a promising tool for preoperative prediction of LN status in patients with GBC.

## Introduction

Gallbladder cancer (GBC) is the most common malignant tumor of the biliary tract, accounting for 80%–95% of biliary tract cancer cases in the world and is ranked the sixth among gastrointestinal cancers (1). GBC is commonly detected in patients along with cholecystitis; however, early diagnosis is challenging owing to quiet symptoms and limited imaging methods. Poor diagnosis results in low median overall survival and low 5-year survival rate (2, 3). Although surgery is associated with poor prognosis, it is the primary approach for treatment of patients with GBC (4). However, only 20%–30% of patients diagnosed in the clinic can undergo radical resection and the postoperative recurrence rate reaches 50%–70% owing to late diagnosis (5).

Lymph node metastasis (LNM) is the most important factor in clinical staging of GBC. Patients with positive LNM are classified as stage IIIb, based on the 8th edition of the American Joint Committee on Cancer (AJCC) gallbladder cancer staging system (GBC). Stage IIIb indicates worse prognosis compared with prognosis of earlier stages (6–8). Radical cholecystectomy including expanded systemic lymph node (LN) dissection is recommended to improve surgical outcome (5, 9). However, not all patients can benefit from radical lymphadenectomy. Previous studies reported that extended radical resection should not be conducted in patients with negative LNM because it may cause serious postoperative complications (10, 11). Conversely, patients diagnosed with extensive LNM can choose neoadjuvant therapy or other conversion treatments as the first choice to improve tumor resectability. Therefore, studies should develop methods for accurately predicting LNM status for patients with GBC before making treatment decision.

Computed tomography (CT) is a widely used imaging method; however, it is limited in discovering positive LNM and the diagnostic rate is approximately 24% (12). Most swollen LN caused probably by cholecystitis or biliary obstruction (13) can be detected through CT examination whereas positive LNs < 1 mm cannot be detected by the naked eye (14). Therefore, it is difficult for surgeons to distinguish LNM with the assistance of the conventional imaging method (15).

Radiomics technology, a product of artificial intelligence, can extract several imaging features from quantitative medical images (16, 17). Radiomics technology is a powerful tool for predicting LNM in different cancers (18–24). However, studies have not explored prediction of LNM based on radiomics technology in GBC.

The aim of the current study was to develop and validate a clinical-radiomics nomogram based on CT images that incorporate the radiomics signature and clinical pathological characteristics to quantitatively predict LNM of GBC.

## Materials and Methods

### Patients

**Figure 1:**
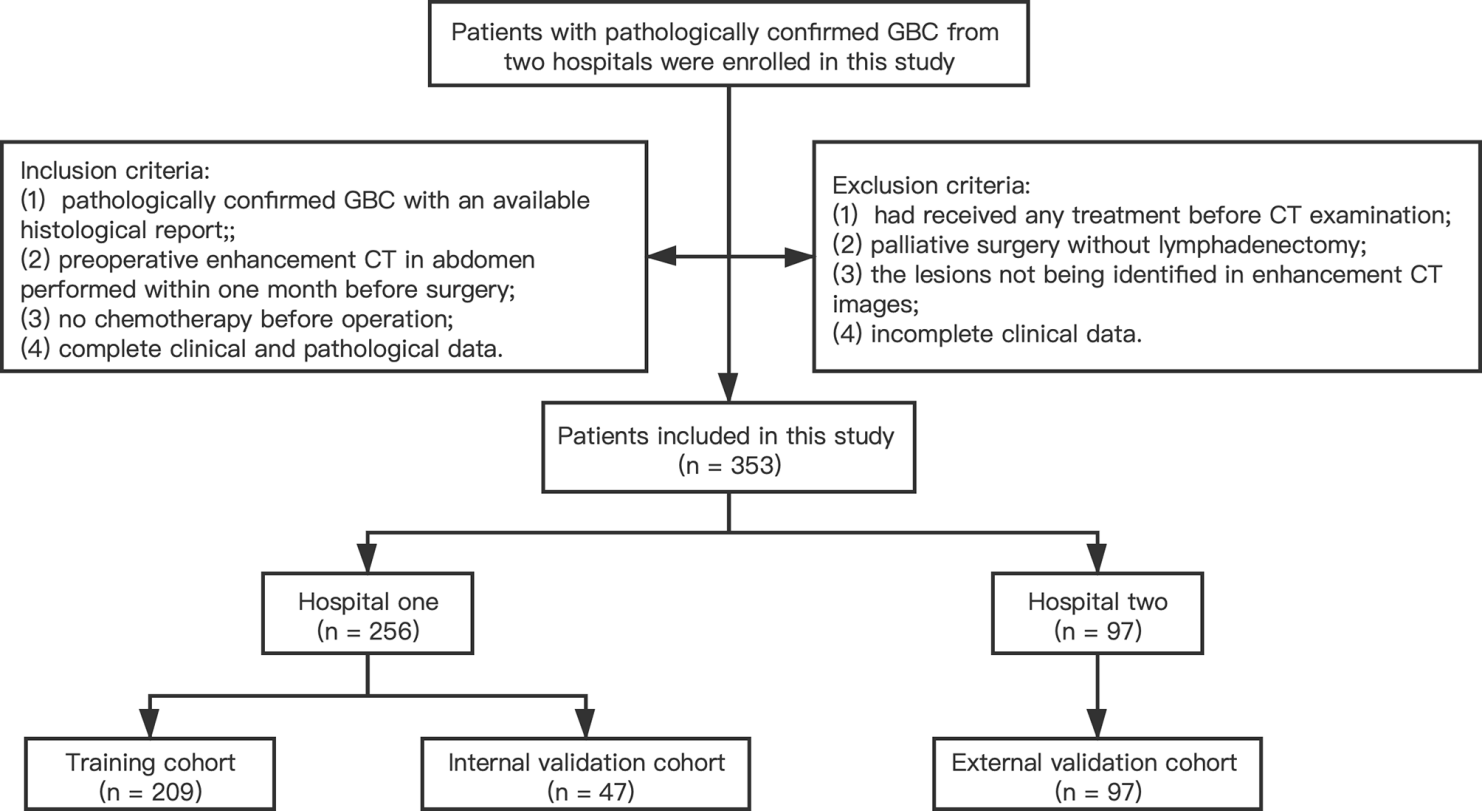
Recruitment pathway of patients.

A total of 353 patients with GBC from two medical centers were enrolled to the current study. The training cohort and internal validation cohort comprised 209 patients and 47 patients with radical cholecystectomy recruited from the Second Affiliated Hospital Zhejiang University School of Medicine (Zhejiang, China) between January 2013 and December 2018, and between January 2019 and December 2020, respectively. The external validation cohort comprised 97 patients with radical cholecystectomy enrolled from the First Affiliated Hospital Zhejiang University School of Medicine (Zhejiang, China) between January 2013 and December 2018 following the same enrollment procedures. Inclusion criteria were as follows: (1) pathologically confirmed GBC with an available histological report; (2) preoperative enhancement CT in abdomen performed within 1 month before surgery; (3) no chemotherapy or other treatment before operation; and (4) complete clinical and pathological data. Exclusion criteria were as follows: (1) had received any treatment (radiotherapy, chemotherapy, or immunotherapy) before CT examination; (2) patients that have undergone palliative surgery without lymphadenectomy; (3) lesions that cannot be identified in enhancement CT images; and (4) incomplete clinical data. A flowchart for patient recruitment is shown in **Figure 1**. The ethics committees of two hospitals approved this retrospective analysis and waived the requirement for informed consent.

**Figure 2:**
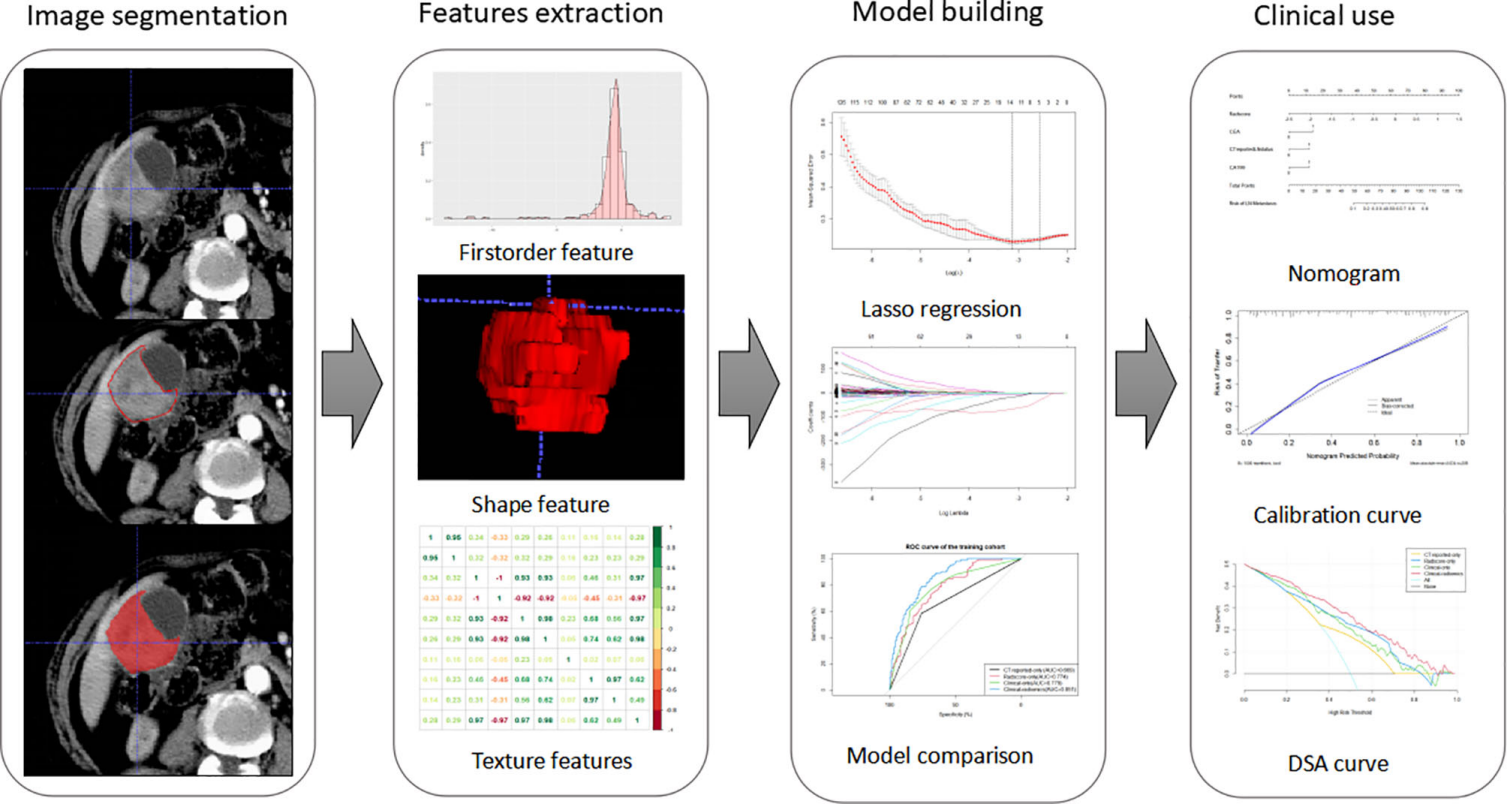
Workflow of this study. Tumors are segmented manually on axial portal venous-phase CT section. Radiomics features were extracted from each CT images. LASSO regression was used to select radiomics features. The radiomics signature is constructed by a linear combination of selected features. Then, we built four models and compared their prediction performances with ROC curves. As a result, a clinical-radiomics nomogram was developed based on the best model. Calibration curves and DSA curves were used to evaluate the clinical utility of the nomogram.

Baseline clinical information, including age, gender, tumor markers, inflammatory indicators, presence of gallstones, symptoms, and CT report, was obtained from electronic medical records. Details on clinical characteristics are presented in **Table 1**. The gold standard for LNM was pathologically evaluated after surgery. Positive LNs were determined based on preoperative CT images by experienced radiologists, following the 8th AJCC TNM staging system. A flow diagram showing the study procedures is shown in **Figure 2**.

**Table 1:** The clinical characteristics of the training cohort and the validation cohorts.

Characteristics		Training cohort (*n* = 209)	Internal validation cohort (*n* = 47)	External validation cohort (*n* = 97)
Gender	Male	66 (31.6%)	13 (27.7%)	28 (28.9%)
	Female	143 (68.4%)	34 (72.3%)	69 (71.1%)
Age	<60 years	66 (31.6%)	14 (29.8%)	32 (33.0%)
	≥60 years	143 (68.4%)	33 (70.2%)	65 (67.0%)
Gallstone	Yes	103 (49.3%)	15 (31.9%)	49 (50.5%)
	No	106 (50.7%)	32 (68.1%)	48 (49.5%)
Cholecystitis	Yes	132 (63.2%)	35 (37.1%)	61 (62.9%)
	No	77 (36.8%)	12 (25.5%)	36 (37.1%)
Jaundice	Yes	26 (12.4%)	7 (14.9%)	9 (9.3%)
	No	183 (87.6%)	40 (85.1%)	88 (90.7%)
NLR	Normal	105 (50.2%)	25 (53.2%)	48 (49.5%)
	Elevated	104 (49.8%)	22 (46.8%)	49 (50.5%)
PLR	Normal	106 (50.7%)	28 (59.6%)	47 (48.5%)
	Elevated	103 (49.3%)	19 (40.4%)	50 (51.5%)
ALT (U/L)	Normal	140 (67.0%)	31 (66.0%)	71 (73.2%)
	Elevated	69 (33.0%)	16 (34.0%)	26 (26.8%)
AST (U/L)	Normal	150 (71.8%)	34 (72.3%)	76 (78.4%)
	Elevated	59 (28.2%)	13 (27.7%)	21 (21.6%)
AFP (ng/ml)	Normal	11 (5.3%)	3 (6.4%)	5 (5.2%)
	Elevated	198 (94.7%)	44 (93.6%)	92 (94.8%)
CEA (ng/ml)	Normal	60 (28.7%)	14 (29.8%)	22 (22.7%)
	Elevated	149 (71.3%)	33 (70.2%)	75 (77.3%)
CA125 (ng/ml)	Normal	55 (26.3%)	14 (29.8%)	18 (18.6%)
	Elevated	154 (73.7%)	33 (70.2%)	79 (81.4%)
CA199 (ng/ml)	Normal	101 (48.3%)	24 (51.1%)	44 (45.4%)
	Elevated	108 (51.7%)	23 (48.9%)	53 (54.6%)
CT reported LN status	Positive	84 (40.2%)	15 (31.9%)	34 (35.1%)
	Negative	125 (59.8%)	32 (68.1%)	63 (64.9%)
Radscore		−0.143	0.294	−0.244
		(−0.033 to 0.234)	(−0.208 to 0.243)	(−0.019 to 0.220)

NLR, neutrophil-to-lymphocyte ratio; PLR, platelet-to-lymphocyte ratio; ALT, alanine aminotransferase; AST, aspartate aminotransferase; CEA, carcinoembryonic antigen; CA125, carbohydrate antigen 125; CA19-9, carbohydrate antigen 19-9.

### Acquisition

Abdominal CT enhancement examinations were performed on all patients within 1 month before the operation. Enhanced CT scan in the first hospital was performed using three CT scanners, including a 64-slice CT, a 256-slice CT (Philips Healthcare), and a 16-slice CT (Toshiba Medical System). Contrast-enhanced CT scan in the second hospital was performed using two CT scanners, including a 40-slice CT (Siemens AG) and a 320-slice CT (Toshiba Medical Systems). CT scan parameters of the two hospitals were uniform and included the following: tube voltage at 120 kVp, tube current ranging from 125 to 300 mAs, pitch ranging from 0.6 to 1.25 mm, slice thickness ranging from 3 to 5 mm, and reconstruction interval from 3 to 5 mm. A high-pressure syringe was used to administer the non-ionic contrast agent Ultravist (Bayer Schering Pharma) (1.5 ml/kg) at a rate of 3.0 ml/s. CT scans of the arterial phase and portal vein phase were performed at 25 to 35 s and 55 to 75 s after administration of the non-ionic contrast agent.

### Image Segmentation and Extraction of Features

Regions of interest (ROIs) were manually segmented slice by slice around the lesions using an open-source imaging platform (ITK-SNAP, version 3.6.0). The portal venous phase was selected for image segmentation because it indicates the tumor boundary more accurately. Voxel size of the images was resampled to a normalized 1 × 1 × 1 mm^3^ to eliminate the pixel difference of the images, and voxel size and all the tumor regions were quantified as 64-gray levels to normalize the inhomogeneity of datasets due to variable tube voltages. Features were extracted from each segmented ROI and were divided into non-textual features and textural features using an in-house Python script (Pyradiomics version: stable; http://github.com/Radiomics/pyradiomics) (25).

Reproducibility of intra-observer and inter-observer agreement for ROI drawing was evaluated using 20 randomly chosen samples drawn from portal venous phase images by two radiologists blinded from patients’ characteristics. A radiologist (reader 1) with 20 years of abdominal imaging experience and a surgeon (reader 2) with 15 years of surgical experience reviewed all CT scans to explore characteristics of each image. Reader 1 performed ROI drawing and feature extraction twice in a 2-week period, following a similar procedure to assess intra-observer reproducibility. In addition, reader 2 independently carried out the same procedure. Then, inter-observer agreement was assessed by comparing the results with the radiomics features extracted from the first ROI between two readers. Intra-observer and inter-observer agreement were assessed by intraclass correlation coefficient (ICC). An ICC > 0.75 represented satisfactory agreement.

### Radiomic Feature Selection and Radscore Building

**Figure 3:**
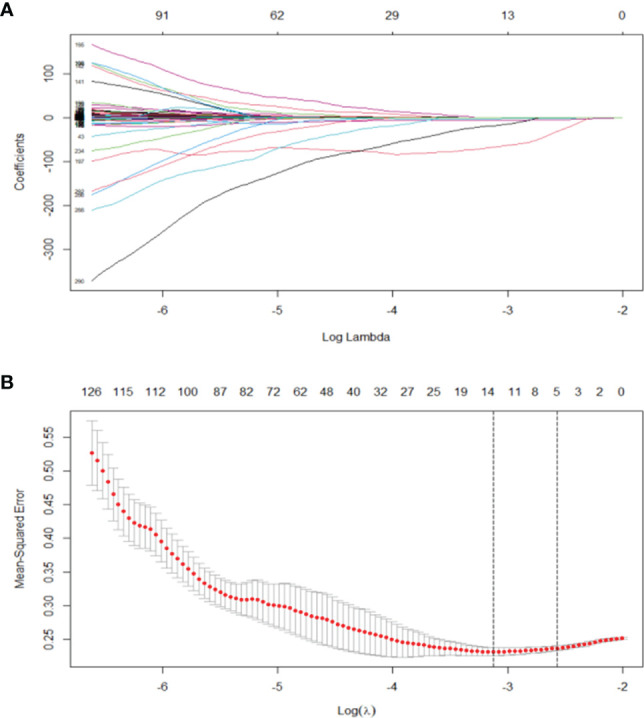
**(A)** LASSO, least absolute shrinkage and selection operator. A total of 293 radiomics features. **(B)** Tuning parameter (*λ*) selection in the LASSO model used 10 cross-validation *via* minimum criteria (the 1-SE criteria). Vertical lines were drawn at the optimal values. The optimal *λ* (*λ* = 0.044) resulted in 14 features with nonzero coefficients.

Least absolute shrinkage and selection operator (LASSO) algorithm was used to determine penalty coefficient with 10-fold cross-validation, which was then used to select optimal features from the training cohort (26, 27). A radiomics score (Radscore) of each patient was calculated by a linear combination of selected features, which were weighted based on their respective coefficients (**Figure 3**). More details on LASSO regression and radiomics features are presented in the **Supplementary Material**.

### Development of CT Reported-Only, Clinical, and Clinical-Radiomics Model

Univariate logistic regression analysis was used to explore the relationship between LNM status and each clinical parameter, biomarkers, and Radscore in the training cohort. Significantly correlated clinical risk factors were then used for stepwise multivariate logistic regression analysis to build the clinical-only model. CT-reported LN status, an independent risk factor, was used to build a CT reported-only model.

Moreover, clinical risk factors of the clinical-only model and Radscore were used for multiple logistic regression analysis to build the clinical-radiomics model.

### Model Comparison and Nomogram Development

Each model was selected based on the minimal Akaike’s information criterion (AIC) to determine the best diagnostic model. Area under the curve (AUC) was used to determine prediction accuracy of the four models in the training and validation cohort. Sensitivity, specificity, and accuracy of each model in the primary and validation cohort were calculated. A nomogram was then built based on the most effective model for LNM prediction of GBC. A calibration curve was plotted to evaluate both discrimination and calibration of the best nomogram.

### Clinical Use of the Nomogram

To explore the clinical utility of the nomogram, decision curve analysis (DCA) was performed based on four models to determine the utility of the nomogram for a range of threshold probabilities.

### Statistical Analysis

Statistical analyses were conducted using SPSS software (version 21.0) and R software (version 4.0.0). Continuous variables were compared using Mann–Whitney *U* test, whereas category variables were compared using Chi-squared or Fisher exact tests. Univariate and multivariate Cox regression analyses were performed to determine predictors of LN. Variables with *p*-value < 0.05 in univariate analysis were selected for multivariate analysis. LASSO regression analysis was performed using the “glmnet” package in R software version 4.0.0. “pROC” package was used to plot the ROC curve. Nomogram construction and calibration plots were generated using “rms” package in R. DCA was performed using the “dca.R” package. A two-sided *p* < 0.05 was considered statistically significant.

## Results

### Clinical Characteristics

Patient characteristics in the training and validation cohorts are presented in **Table 1**. Analysis showed no significant differences in clinicopathological characteristics between the three groups.

### Feature Selection and Radscore Development

A total of 293 radiomics features based on the training cohort were reduced to 14 potential predictors using LASSO regression analysis (**Figure 2**). A radiomics score (Radscore) was then calculated using the formula presented in the Supplementary material. Findings from univariate logistic regression analysis showed that the Radscore (OR = 9.610; 95% CI: 4.579–20.168, *p* < 0.001) was an independent variable for prediction of LNM in the training cohort (**Table 2**).

**Table 2:** Univariate and multivariate logistic regression analysis for LN metastasis in the primary cohort.

Characteristics	Univariate analysis		Multivariate analysis			
			Clinical model		Clinical-radiomics model	
	OR (95% CI)	*P*	OR (95% CI)	*P*	OR (95% CI)	*p*
Gender	1.301 (0.723–2.343)	0.380	NA	NA	NA	NA
Age	0.762 (0.425–1.368)	0.363	NA	NA	NA	NA
Gallstone	0.776 (0.450–1.337)	0.361	NA	NA	NA	NA
Cholecystitis	0.929 (0.529–1.631)	0.797	NA	NA	NA	NA
Jaundice	1.643 (0.716–3.770)	0.241	NA	NA	NA	NA
NLR	2.138 (1.229–3.718)	0.007	1.207 (0.590–2.467)	0.607	1.044 (0.484–2.252)	0.913
PLR	2.622 (1.498–4.590)	0.001	1.963 (0.977–3.944)	0.058	1.888 (0.900–3.962)	0.093
ALT	1.497 (0.839–2.672)	0.172	NA	NA	NA	NA
AST	2.014 (1.092–3.715)	0.025	1.403 (0.680–2.897)	0.360	1.299 (0.579–2.912)	0.525
AFP	3.200 (0.825–12.419)	0.093	NA	NA	NA	NA
CEA	4.323 (2.251–8.303)	<0.001	2.898 (1.418–5.921)	0.004	3.183 (1.423–7.123)	0.005
CA125	3.925 (2.016–7.643)	<0.001	1.872 (0.857–4.087)	0.116	1.557 (0.661–3.672)	0.311
CA199	4.895 (2.722–8.800)	<0.001	3.597 (1.921- 6.733)	0.001	2.230 (1.074–4.632)	0.031
CT reported LN status	4.325 (2.396–7.809)	<0.001	2.962 (1.557–5.635)	0.010	2.261 (1.075–4.755)	0.031
Radscore	9.610 (4.579–20.168)	<0.001	NA	NA	6.645 (3.025–14.597)	<0.001

OR, odds ratio; NLR, neutrophil-to-lymphocyte ratio; PLR, platelet-to-lymphocyte ratio; ALT, alanine aminotransferase; AST, aspartate aminotransferase; CEA, carcinoembryonic antigen; CA125, carbohydrate antigen 125; CA19-9, carbohydrate antigen 19-9, NA, not available.

### Development of the Clinical Model and CT Reported-Only Model

Results of the univariate analysis and the multivariate regression analysis using training cohort are shown in **Table 2**. Univariate analysis of the training cohort identified NLR, PLR, aspartate aminotransferase (AST), carcinoembryonic antigen (CEA), carcinoembryonic antigen 125 (CA125), carcinoembryonic antigen 199 (CA199), and CT reported LN status as statistically significant risk factors (*p* < 0.05) (**Table 2**). Statistically significant variables selected from the univariate analysis were used for binary multiple logistic regression, and the findings showed that CEA (OR = 2.898; 95% CI: 1.418–5.921; *p* = 0.004), CA199 (OR = 3.597; 95% CI: 1.921–6.733; *p* < 0.001), and CT reported LN status (OR = 2.962; 95% CI: 1.557–5.635; *p* = 0.001) were independent risk factors of LNM in the training cohort. The three independent risk factors based on the logistic multivariate regression analysis were used for construction of the clinical model (**Table 3**). In addition, CT reported LN status identified as an independent variable in univariate analysis was used to build CT reported-only model (OR = 4.325; 95% CI: 2.396–7.809; *p* < 0.001).

**Table 3:** Comparison of four models by multivariate logistic regression analysis.

Variable	CT reported-only model			Radscore-only model			Clinical model			Clinical-radiomics model		
	OR (95% CI)	*P*	AIC	OR (95% CI)	*P*	AIC	OR (95% CI)	*P*	AIC	OR (95% CI)	*p*	AIC
CEA	NA	NA	267.68	NA	NA	237.31	2.898 (1.418–5.921)	0.004	242.27	3.122 (1.426–6.835)	0.004	209.18
CA199	NA	NA		NA	NA		3.597 (1.921–6.733)	<0.001		2.592 (1.288–5.215)	0.008	
CT reported LN status	4.325 (2.396–7.809)	<0.001		NA	NA		2.962 (1.557–5.635)	0.001		2.597 (1.278–5.279)	0.008	
Radscore	NA	NA		9.610 (4.579–20.168)	<0.001		NA	NA		7.415 (3.384–16.246)	<0.001	

AIC, Akaike’s information criterion; OR, odds ratio; CI, confidence interval, NA, not available.

### Development of the Clinical-Radiomics Model

Eight factors, namely, NLR, PLR, AST, CEA, CA125, CA199, CT reported LN status, and Radscore, were used for binary multivariate logistic regression analysis (**Table 2**). The findings showed that CEA (OR = 3.122; 95% CI: 1.426–6.835; *p* = 0.004), CA199 (OR = 2.592; 95% CI: 1.288–5.215; *p* = 0.008), CT reported LN status (OR = 2.597; 95% CI: 1.278–5.279; *p* = 0.008), and Radscore (OR = 7.415; 95% CI: 3.384–16.246; *p* < 0.001) were significant predictors of LNM; thus, they were used to build a clinical-radiomics model for LNM (**Table 3**). Notably, Radscore was the dominant factor affecting prediction of LNM in the clinical-radiomics model.

### Model Comparison and Validation of the Nomogram

AIC was used to determine the goodness of model fitting. Comparison was performed for the clinical model (AIC = 242.27), the Radscore-only model (AIC = 237.31), the CT reported-only model (AIC =267.68), and the clinical-radiomics model (AIC = 209.18). Notably, the clinical-radiomics model had the lowest AIC value (AIC = 209.18) and was identified as the best model.

ROC curves were used to evaluate the accuracy and predictive value of the four models (**Table 4** and **Figure 5A**). In the training cohort, the clinical-radiomics model showed the highest discrimination between LN positive and negative cases, with an AUC of 0.851 (95% CI, 0.801–0.901). The AUC value of the clinical-radiomics model was significantly higher compared with that of the clinical model (AUC 0.779; 95% CI, 0.715–0.842; *p* = 0.001), Radscore-only model (AUC 0.774; 95% CI, 0.712–0.836; *p* = 0.001), and CT reported-only model (AUC 0.669; 95% CI, 0.595–0.743; *p* < 0.001). In the internal validation cohort and external validation cohort, the radiomics model showed the highest AUC of 0.824 (95% CI, 0.741–0.908) and 0.819 (95% CI, 0.645–0.993), respectively. The clinical-radiomics model showed the best accuracy for prediction efficiency of LNM in the training cohort (sensitivity: 86.7%; specificity: 67.6%; accuracy: 78.0%), internal validation cohort (sensitivity: 73.3%; specificity: 100%; accuracy: 91.5%), and external validation cohort (sensitivity: 78.7%; specificity: 80%; accuracy: 79.4%) (**Table 4**).

**Table 4:** Accuracy and predictive value between four models.

Models	Training cohort				Internal validation cohort				External validation cohort			
	Sensitivity	Specificity	Accuracy	AUC (95% CI)	Sensitivity	Specificity	Accuracy	AUC (95% CI)	Sensitivity	Specificity	Accuracy	AUC (95% CI)
CT reported-only	(57/98) 58.2%	(84/111) 75.7%	(141/209) 67.5%	0.669 (0.595–0.743)	(6/15) 40%	(23/32) 71.9%	(29/47) 61.7%	0.559 (0.379–0.739)	(26/47) 55.3%	(42/50) 84.0%	(68/97) 70.1%	0.697 (0.590–0.803)
Radscore-only	(80/98) 81.6%	(67/111) 60.4%	(147/209) 70.3%	0.774 (0.712–0.836)	(12/15) 80.0%	(30/32) 93.8%	(42/47) 89.4%	0.763 (0.668–0.857)	(27/47) 57.4%	(42/50) 84.0%	(69/97) 71.1%	0.763 (0.668–0.857)
Clinical	(75/98) 76.5%	(76/111) 68.5%	(151/209) 72.2%	0.779 (0.715–0.842)	(12/15) 80.0%	(18/32) 56.2%	(30/47) 68.1%	0.731 (0.631–0.832)	(37/47) 78.7%	(31/50) 62.0%	(68/97) 70.1%	0.731 (0.631–0.832)
Clinical-radiomics	(85/98) 86.7%	(75/111) 67.6%	(160/209) 78.0%	0.851 (0.801–0.901)	(11/15) 73.3%	(32/32) 100%	(77/97) 91.5%	0.819 (0.645–0.993)	(37/47) 78.7%	(40/50) 80%	(77/97) 79.4%	0.824 (0.741–0.908)

AUC, area under the curve.

**Figure 4:**
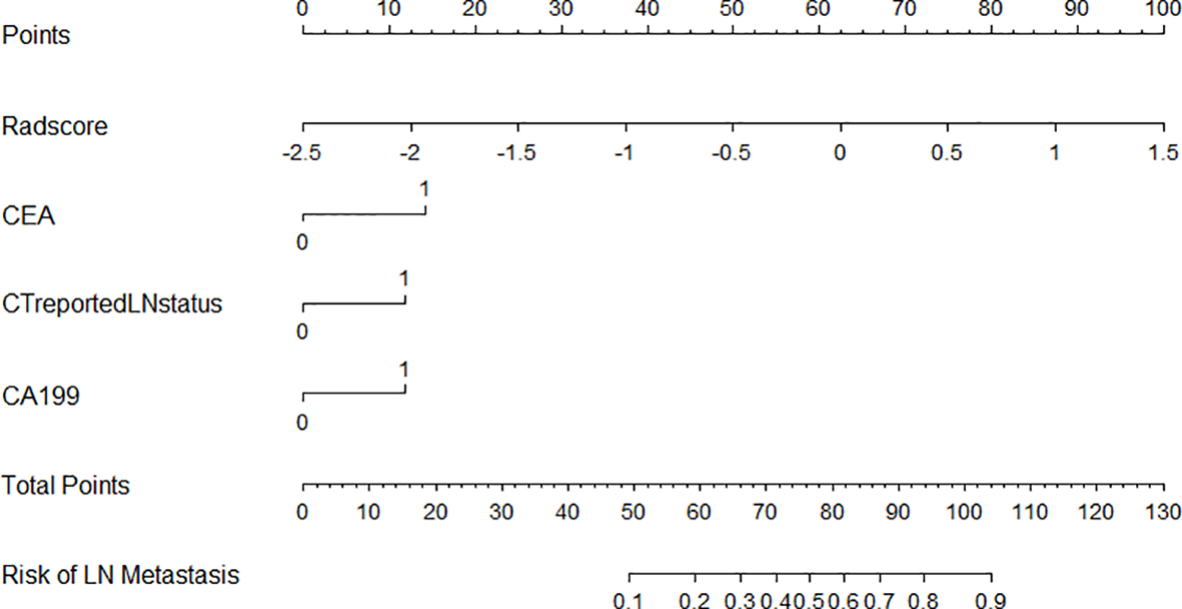
Developed clinical-radiomics nomogram. The clinical-radiomics nomogram was developed including Radscore, CEA, CA199, and CT reported LN status in the training group.

**Figure 5:**
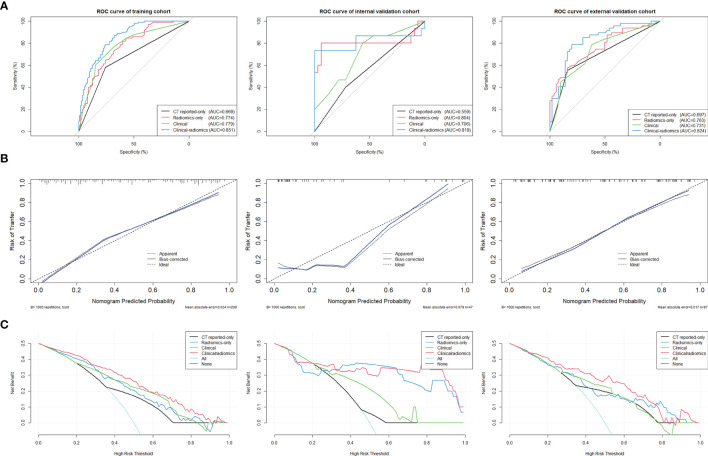
**(A)** ROC curves of the clinical-radiomics model, the clinical model, the Radscore-only model, and the CT reported-only model were shown in the training cohort, internal validation cohort, and external validation cohort, respectively. **(B)** Calibration curves of the clinical-radiomics nomogram for predicting LNM between prediction and actual LN status in the training cohort, internal validation cohort, and external validation cohort. The 45° straight line represents an ideal model perfectly calibrated with an outcome. A closer distance between two curves indicates higher accuracy. **(C)** Decision curve analysis for the clinical-radiomics nomogram, the clinical model, the Radscore-only model, and the CT reported-only model in both three cohorts. The *y*-axis measures the net benefit. The red line represents the clinical-radiomics nomogram. The blue line represents the Radscore-only model. The green line represents the clinical model. The yellow line represents CT reported-only model. The azure line represents the assumption that all patients had LNM. The black line represents the assumption that no patients had LNM.

The clinical-radiomics model showed the best discrimination and predictive ability among the four models. Therefore, a clinical-radiomics nomogram was successfully developed based on the clinical-radiomics model (**Figure 4**). A calibration curve of the clinical-radiomics nomogram for the probability of LNM showed good consistency between prediction and actual LN status in the three cohorts (**Figure 5B**).

### Clinical Application

DCA curves for the clinical-radiomics model, the clinical model, the Radscore-only model, and the CT reported-only model in both the training and validation cohorts are shown in **Figure 5C**. The threshold probability of the clinical-radiomics nomogram was more than 10%, which was better compared with the other three models in predicting LNM. The combined nomogram including radiomics signature showed the maximum clinical utility at almost all threshold probabilities.

## Discussion

To the best of our knowledge, this is the first study to develop a clinical-radiomics nomogram based on radiomics technology for preoperative prediction of LNM in patients with GBC. The clinical-radiomics model incorporated radiomics signature and three clinical variables including CEA, CA199, and CT reported LN status. The Radscore was calculated based on the radiomics signature and the findings showed that it was an independent factor in predicting LNM. Addition of radiomic analysis significantly improved the predictive accuracy of the combined model. The findings indicated that the clinical-radiomics nomogram is effective for preoperative prediction of LNM in GBC and can help in making clinical decisions during GBC treatment.

Previous studies explored non-invasive methods that can quantitatively predict preoperative LNM in GBC. Several conventional imaging examinations such as contrast-enhanced CT, MRI, and 18-FDG PET/CT are used to determine the LN status and LN larger than 1 cm in diameter is considered a standard for positive LNM in these examinations. However, swollen LN can be caused by biliary inflammation or biliary obstruction. Petrowsky et al. reported that the accuracy of enhanced CT and PET/CT for regional LNM prediction was 24% *vs.* 12% (12). A meta-analysis of 14 institutes reported that although MRI is effective in predicting LNM of GBC, it is challenging to detect LNM less than 1 cm (28). Fine needle aspiration for pathological biopsy is the gold standard for preoperative diagnosis; however, it can only be applied to a limited range of patients. The method is associated with severe complications such as bleeding, tumor dissemination, and lymphatic fistula owing to the use of fine needle aspiration. Several studies report that inflammation and metabolites in tumor areas of the biliary system may cause LN hyperplasia (29–31). Therefore, the current study established a clinical model including multiple parameters for comparison. The findings showed that NLR, PLR, AST, and CA125 were independent predictive factors for LNM in univariate analysis. However, analysis showed no significant correlation between these markers with LNM using multivariate logistics regression analysis. The clinical model build using CEA, CA199, and CT reported LN status showed higher prediction value compared with CT reported-only model; however, its overall accuracy was still unsatisfactory. Conventional imaging methods are based on morphological criteria and serum biomarkers and do not meet the clinical need for quantitative and accurate diagnosis.

On the contrary, radiomics technology is a quantitative method and thus it is effective in preoperative LN assessment. Several studies report that radiomics can be correlated with tumor gene characteristics and protein phenotypes to predict the biological behavior of tumors (32, 33). Metastatic LN and non-metastatic LN present different biological behaviors (10). Studies report that a model established based on radiomics has great potential in predicting LNM of malignant tumors (21–23). In the current study, a CT-based radiomics model composed of 14 radiomics signatures was established using LASSO regression. The Radscore established included shape, first-order, and textural features. Radiomics analysis of CT images can help distinguish between positive LNM and negative LNM for gastric cancer and colorectal cancer (20, 21). The findings of the current study showed that the radiomics model based on morphological features and texture features has better predictive accuracy and goodness of fit for LNM compared with the single CT reported-only model (AUC = 0.781 *vs.* AUC = 0.669; AIC 237.31 to 267.68).

For better clinical application, a clinical-radiomics nomogram was established that integrated Radscore and clinical variables. This comprehensive nomogram showed higher accuracy and discrimination of the LNM in GBC compared with the other three models. Calibration curves and DCA curves showed that the nomogram had high consistency and potential clinical applicability in the two medical centers. The clinical-radiomics nomogram can be used effectively to determine the possibility of surgical R0 resection, thus assisting in preoperative treatment decision-making. GBC patients suspected of positive LNM based on conventional imaging reports can use the nomogram to reconfirm their LN status. In addition, surgeons can use the nomogram to accurately assess the necessity for LN resection before surgery to benefit patients with actual negative LNM, thus reducing complications and hospitalization costs.

The current study had some limitations. Firstly, genetic diagnosis related to progress of GBC may provide more value in the diagnosis of LNM through development of radiogenomic biomarkers. Further studies that include genotypes to new predictive models to improve the model’s diagnostic accuracy should be conducted. Secondly, the data used to build the model in the current study were obtained from two large-scale medical centers in a region that may lead to data bias. Therefore, studies should include more patients from multiple centers as a verification cohort to verify the clinical applicability and robustness of the nomogram.

In summary, the clinical-radiomics nomogram reported in the current study can be used as a non-invasive biomarker for preoperative prediction of LNM in GBC patients. The findings show that the model is useful in clinical decision-making and can improve the survival outcome of patients with GBC.

## Data Availability Statement

The raw data supporting the conclusions of this article will be made available by the authors, without undue reservation.

## Ethics Statement

The ethics committees of two hospitals approved this retrospective analysis and waived the requirement for informed consent.

## Author Contributions

XLiu collected CT images and clinical data, and drafted the original manuscript. XLia analyzed the data and revised the manuscript. LR and SY provided the idea, designed the research, segmented the images, and revised the manuscript. All authors contributed to the article and approved the submitted version.

## Funding

This project was supported by the National Natural Science Foundation of China (No. 81572975), the Key Research and Development Project of the Science and Technology Department of Zhejiang, China (No. 2015C03053), and the Zhejiang Provincial Program for the Cultivation of High-level Innovative Health talents.

## Conflict of Interest

The authors declare that the research was conducted in the absence of any commercial or financial relationships that could be construed as a potential conflict of interest.

## Publisher’s Note

All claims expressed in this article are solely those of the authors and do not necessarily represent those of their affiliated organizations, or those of the publisher, the editors and the reviewers. Any product that may be evaluated in this article, or claim that may be made by its manufacturer, is not guaranteed or endorsed by the publisher.
