# Supplementary material for: A Clinical-Radiomics Nomogram for Preoperative Prediction of Lymph Node Metastasis in Gallbladder Cancer
Source: Front Oncol. 2021 Sep 22;11:633852. doi: 10.3389/fonc.2021.633852 (PMC8493033; doi:10.3389/fonc.2021.633852)
Supplement: Supplementary file 1 [file DataSheet_1.docx]

Supplementary Material

1. **Supplementary Data**

## 1.1 Details of clinical features:

1. patient's gender:male and female;
2. patient's age(during surgery): < 60 y and ≥ 60 y;
3. history of gallstones: yes (have a history of gallbladder stones) ; no (without history of gallbladder stones);
4. history of cholecystitis: yes (have a history of cholecystitis); no (without history of cholecystitis);
5. history of jaundice: yes (have a history of jaundice); no (without history of jaundice);
6. baseline level of neutrophil-to-lymphocyte ratio (NLR) (first tested before surgery): the median of NLR as cut-off values was 2.52, with 0～2.52 ng / ml for normal and ≥ 2.52 for elevated;
7. baseline level of platelet-to-lymphocyte ratio (PLR) (first tested before surgery) : the median of PLR as cut-off values was 145.88, with 0～145.88 ng / ml for normal and ≥ 145.88 for elevated;
8. baseline level of alanine (ALT) (first tested before surgery): with 0～35 U/L for normal and ≥ 35 U/Ll for elevated in hospital one; with 0～45 U / L for normal and ≥ 45 U / Ll for elevated in hospital two;
9. baseline level of aspartate aminotransferase (AST) (first tested before surgery): with 0～40 U / L for normal and ≥ 40 U / L for elevated in hospital one; with 0～35 U / L for normal and ≥ 35 U / L for elevated in hospital two;aaaa
10. baseline level of carcinoembryonic antigen (CEA) (first tested before surgery): with 0～5 ng / mL for normal and ≥ 5 ng / mL for elevated in both two hospitals;
11. baseline level of carbohydrate antigen 199 (CA 199) (first tested before surgery): with 0～37 U / mL for normal and ≥37 U / mL for elevated in both two hospitals;
12. baseline level of carbohydrate antigen 125 (CA 125) (first tested before surgery): with 0～35 U / mL for normal and ≥ 35 U / mL for elevated in both two hospitals;
13. baseline level of alpha-fetoprotein (AFP) (first tested before surgery): with 0～20 ng / mL for normal and ≥ 35 ng / mL for elevated in both two hospitals;

**1.2 Radiomics feature extraction methodology**

Features were extracted using Python (Pyradiomics version: stable; <http://github.com/Radiomics/pyradiomics).> A total of 293 features were extracted , including 14 shape features, 54 firstorder features, 72 glcm features, 42 gldm features, 48 glrlm features, 48 glszm features, 15 ngtdm features from each CT images. **Table S1** listed the extracted radiomics features. The format of the original image was DICOM.

**1.3 The least absolute shrinkage and selection operator (LASSO) algorithm**

We use LASSO regression to select radiomics features related to LNM of GBC patients. LASSO regression is currently the most popular high-dimensional data dimensionality reduction tool, which can selectively put variables into the model to obtain better performance parameters. The degree of LASSO regression complexity adjustment is controlled by the parameter λ. The larger the λ, the greater the penalty for the linear model with more variables, so that a model with fewer variables is finally obtained. We selected the features when λ takes the maximum value to build a radiomics signature (Rad-score).

**1.4 Radscore calculation formula**

**Radscore** =

2.666690

+ 1.736603 * 0.1 * original_shape_Elongation

+ 2.725802 * 0.001* original_shape_Maximum2DDiameterColumn

- 2.538924 * original_shape_Sphericity

+ 1.307822 * 0.01 * original_firstorder_Skewness

+ 1.798748 * 0.01 * logsigma10mm3D_firstorder_Mean

+ 9.425804 * 0.00001 * logsigma10mm3D_firstorder_Median

- 1.581876 * 0.1 * logsigma10mm3D_glrlm_RunEntropy

- 9.450247 * 0.001 * logsigma10mm3D_glszm_SizeZoneNonUniformity

- 5.926717 * logsigma10mm3D_glszm_SizeZoneNonUniformityNormalized

- 6.784287 * 10 * logsigma10mm3D_ngtdm_Coarseness

- 1.981850 * 0.01 * logsigma30mm3D_firstorder_Kurtosis

- 1.773556 * 0.001 * logsigma30mm3D_firstorder_Maximum

- 5.604566 * 0.0000000001 * logsigma30mm3D_glszm_LargeAreaHighGrayLevelEmphasis

- 1.814657 * 10 * logsigma30mm3D_ngtdm_Coarseness

# 2. Supplementary Table

| **Table S1\| Radiomics features extracted from each CT images** | | | |
| --- | --- | --- | --- |
| shape | original |  |  |
|  | original_shape_Elongation |  |  |
|  | original_shape_Flatness |  |  |
|  | original_shape_LeastAxisLength |  |  |
|  | original_shape_MajorAxisLength |  |  |
|  | original_shape_Maximum2DDiameterColumn |  |  |
|  | original_shape_Maximum2DDiameterRow |  |  |
|  | original_shape_Maximum2DDiameterSlice |  |  |
|  | original_shape_Maximum3DDiameter |  |  |
|  | original_shape_MeshVolume |  |  |
|  | original_shape_MinorAxisLength |  |  |
|  | original_shape_Sphericity |  |  |
|  | original_shape_SurfaceArea |  |  |
|  | original_shape_SurfaceVolumeRatio |  |  |
|  | original_shape_VoxelVolume |  |  |
|  |  |  |  |
| firstorder | original | logsigma10mm3D | logsigma30mm3D |
|  | original_firstorder_10Percentile | logsigma10mm3D_firstorder_10Percentile | logsigma30mm3D_firstorder_10Percentile |
|  | original_firstorder_90Percentile | logsigma10mm3D_firstorder_90Percentile | logsigma30mm3D_firstorder_90Percentile |
|  | original_firstorder_Energy | logsigma10mm3D_firstorder_Energy | logsigma30mm3D_firstorder_Energy |
|  | original_firstorder_Entropy | logsigma10mm3D_firstorder_Entropy | logsigma30mm3D_firstorder_Entropy |
|  | original_firstorder_InterquartileRange | logsigma10mm3D_firstorder_InterquartileRange | logsigma30mm3D_firstorder_InterquartileRange |
|  | original_firstorder_Kurtosis | logsigma10mm3D_firstorder_Kurtosis | logsigma30mm3D_firstorder_Kurtosis |
|  | original_firstorder_Maximum | logsigma10mm3D_firstorder_Maximum | logsigma30mm3D_firstorder_Maximum |
|  | original_firstorder_MeanAbsoluteDeviation | logsigma10mm3D_firstorder_MeanAbsoluteDeviation | logsigma30mm3D_firstorder_MeanAbsoluteDeviation |
|  | original_firstorder_Mean | logsigma10mm3D_firstorder_Mean | logsigma30mm3D_firstorder_Mean |
|  | original_firstorder_Median | logsigma10mm3D_firstorder_Median | logsigma30mm3D_firstorder_Median |
|  | original_firstorder_Minimum | logsigma10mm3D_firstorder_Minimum | logsigma30mm3D_firstorder_Minimum |
|  | original_firstorder_Range | logsigma10mm3D_firstorder_Range | logsigma30mm3D_firstorder_Range |
|  | original_firstorder_RobustMeanAbsoluteDeviation | logsigma10mm3D_firstorder_RobustMeanAbsoluteDeviation | logsigma30mm3D_firstorder_RobustMeanAbsoluteDeviation |
|  | original_firstorder_RootMeanSquared | logsigma10mm3D_firstorder_RootMeanSquared | logsigma30mm3D_firstorder_RootMeanSquared |
|  | original_firstorder_Skewness | logsigma10mm3D_firstorder_Skewness | logsigma30mm3D_firstorder_Skewness |
|  | original_firstorder_TotalEnergy | logsigma10mm3D_firstorder_TotalEnergy | logsigma30mm3D_firstorder_TotalEnergy |
|  | original_firstorder_Uniformity | logsigma10mm3D_firstorder_Uniformity | logsigma30mm3D_firstorder_Uniformity |
|  | original_firstorder_Variance | logsigma10mm3D_firstorder_Variance | logsigma30mm3D_firstorder_Variance |
|  |  |  |  |
| glcm | original | logsigma10mm3D | logsigma30mm3D |
|  | original_glcm_Autocorrelation | logsigma10mm3D_glcm_Autocorrelation | logsigma30mm3D_glcm_Autocorrelation |
|  | original_glcm_JointAverage | logsigma10mm3D_glcm_JointAverage | logsigma30mm3D_glcm_JointAverage |
|  | original_glcm_ClusterProminence | logsigma10mm3D_glcm_ClusterProminence | logsigma30mm3D_glcm_ClusterProminence |
|  | original_glcm_ClusterShade | logsigma10mm3D_glcm_ClusterShade | logsigma30mm3D_glcm_ClusterShade |
|  | original_glcm_ClusterTendency | logsigma10mm3D_glcm_ClusterTendency | logsigma30mm3D_glcm_ClusterTendency |
|  | original_glcm_Contrast | logsigma10mm3D_glcm_Contrast | logsigma30mm3D_glcm_Contrast |
|  | original_glcm_Correlation | logsigma10mm3D_glcm_Correlation | logsigma30mm3D_glcm_Correlation |
|  | original_glcm_DifferenceAverage | logsigma10mm3D_glcm_DifferenceAverage | logsigma30mm3D_glcm_DifferenceAverage |
|  | original_glcm_DifferenceEntropy | logsigma10mm3D_glcm_DifferenceEntropy | logsigma30mm3D_glcm_DifferenceEntropy |
|  | original_glcm_DifferenceVariance | logsigma10mm3D_glcm_DifferenceVariance | logsigma30mm3D_glcm_DifferenceVariance |
|  | original_glcm_JointEnergy | logsigma10mm3D_glcm_JointEnergy | logsigma30mm3D_glcm_JointEnergy |
|  | original_glcm_JointEntropy | logsigma10mm3D_glcm_JointEntropy | logsigma30mm3D_glcm_JointEntropy |
|  | original_glcm_Imc1 | logsigma10mm3D_glcm_Imc1 | logsigma30mm3D_glcm_Imc1 |
|  | original_glcm_Imc2 | logsigma10mm3D_glcm_Imc2 | logsigma30mm3D_glcm_Imc2 |
|  | original_glcm_Idm | logsigma10mm3D_glcm_Idm | logsigma30mm3D_glcm_Idm |
|  | original_glcm_Idmn | logsigma10mm3D_glcm_Idmn | logsigma30mm3D_glcm_Idmn |
|  | original_glcm_Id | logsigma10mm3D_glcm_Id | logsigma30mm3D_glcm_Id |
|  | original_glcm_Idn | logsigma10mm3D_glcm_Idn | logsigma30mm3D_glcm_Idn |
|  | original_glcm_InverseVariance | logsigma10mm3D_glcm_InverseVariance | logsigma30mm3D_glcm_InverseVariance |
|  | original_glcm_MaximumProbability | logsigma10mm3D_glcm_MaximumProbability | logsigma30mm3D_glcm_MaximumProbability |
|  | original_glcm_SumEntropy | logsigma10mm3D_glcm_SumEntropy | logsigma30mm3D_glcm_SumEntropy |
|  | original_glcm_SumSquares | logsigma10mm3D_glcm_SumSquares | logsigma30mm3D_glcm_SumSquares |
|  | original_glcm_MCC | logsigma10mm3D_glcm_MCC | logsigma30mm3D_glcm_MCC |
|  | original_glcm_SumAverage | logsigma10mm3D_glcm_SumAverage | logsigma30mm3D_glcm_SumAverage |
|  |  |  |  |
| gldm | original | logsigma10mm3D | logsigma30mm3D |
|  | original_gldm_DependenceEntropy | logsigma10mm3D_gldm_DependenceEntropy | logsigma30mm3D_gldm_DependenceEntropy |
|  | original_gldm_DependenceNonUniformity | logsigma10mm3D_gldm_DependenceNonUniformity | logsigma30mm3D_gldm_DependenceNonUniformity |
|  | original_gldm_DependenceNonUniformityNormalized | logsigma10mm3D_gldm_DependenceNonUniformityNormalized | logsigma30mm3D_gldm_DependenceNonUniformityNormalized |
|  | original_gldm_DependenceVariance | logsigma10mm3D_gldm_DependenceVariance | logsigma30mm3D_gldm_DependenceVariance |
|  | original_gldm_GrayLevelNonUniformity | logsigma10mm3D_gldm_GrayLevelNonUniformity | logsigma30mm3D_gldm_GrayLevelNonUniformity |
|  | original_gldm_GrayLevelVariance | logsigma10mm3D_gldm_GrayLevelVariance | logsigma30mm3D_gldm_GrayLevelVariance |
|  | original_gldm_HighGrayLevelEmphasis | logsigma10mm3D_gldm_HighGrayLevelEmphasis | logsigma30mm3D_gldm_HighGrayLevelEmphasis |
|  | original_gldm_LargeDependenceEmphasis | logsigma10mm3D_gldm_LargeDependenceEmphasis | logsigma30mm3D_gldm_LargeDependenceEmphasis |
|  | original_gldm_LargeDependenceHighGrayLevelEmphasis | logsigma10mm3D_gldm_LargeDependenceHighGrayLevelEmphasis | logsigma30mm3D_gldm_LargeDependenceHighGrayLevelEmphasis |
|  | original_gldm_LargeDependenceLowGrayLevelEmphasis | logsigma10mm3D_gldm_LargeDependenceLowGrayLevelEmphasis | logsigma30mm3D_gldm_LargeDependenceLowGrayLevelEmphasis |
|  | original_gldm_LowGrayLevelEmphasis | logsigma10mm3D_gldm_LowGrayLevelEmphasis | logsigma30mm3D_gldm_LowGrayLevelEmphasis |
|  | original_gldm_SmallDependenceEmphasis | logsigma10mm3D_gldm_SmallDependenceEmphasis | logsigma30mm3D_gldm_SmallDependenceEmphasis |
|  | original_gldm_SmallDependenceHighGrayLevelEmphasis | logsigma10mm3D_gldm_SmallDependenceHighGrayLevelEmphasis | logsigma30mm3D_gldm_SmallDependenceHighGrayLevelEmphasis |
|  | original_gldm_SmallDependenceLowGrayLevelEmphasis | logsigma10mm3D_gldm_SmallDependenceLowGrayLevelEmphasis | logsigma30mm3D_gldm_SmallDependenceLowGrayLevelEmphasis |
|  |  |  |  |
| glrlm | original | logsigma10mm3D | logsigma30mm3D |
|  | original_glrlm_GrayLevelNonUniformity | logsigma10mm3D_glrlm_GrayLevelNonUniformity | logsigma30mm3D_glrlm_GrayLevelNonUniformity |
|  | original_glrlm_GrayLevelNonUniformityNormalized | logsigma10mm3D_glrlm_GrayLevelNonUniformityNormalized | logsigma30mm3D_glrlm_GrayLevelNonUniformityNormalized |
|  | original_glrlm_GrayLevelVariance | logsigma10mm3D_glrlm_GrayLevelVariance | logsigma30mm3D_glrlm_GrayLevelVariance |
|  | original_glrlm_HighGrayLevelRunEmphasis | logsigma10mm3D_glrlm_HighGrayLevelRunEmphasis | logsigma30mm3D_glrlm_HighGrayLevelRunEmphasis |
|  | original_glrlm_LongRunEmphasis | logsigma10mm3D_glrlm_LongRunEmphasis | logsigma30mm3D_glrlm_LongRunEmphasis |
|  | original_glrlm_LongRunHighGrayLevelEmphasis | logsigma10mm3D_glrlm_LongRunHighGrayLevelEmphasis | logsigma30mm3D_glrlm_LongRunHighGrayLevelEmphasis |
|  | original_glrlm_LongRunLowGrayLevelEmphasis | logsigma10mm3D_glrlm_LongRunLowGrayLevelEmphasis | logsigma30mm3D_glrlm_LongRunLowGrayLevelEmphasis |
|  | original_glrlm_LowGrayLevelRunEmphasis | logsigma10mm3D_glrlm_LowGrayLevelRunEmphasis | logsigma30mm3D_glrlm_LowGrayLevelRunEmphasis |
|  | original_glrlm_RunEntropy | logsigma10mm3D_glrlm_RunEntropy | logsigma30mm3D_glrlm_RunEntropy |
|  | original_glrlm_RunLengthNonUniformity | logsigma10mm3D_glrlm_RunLengthNonUniformity | logsigma30mm3D_glrlm_RunLengthNonUniformity |
|  | original_glrlm_RunLengthNonUniformityNormalized | logsigma10mm3D_glrlm_RunLengthNonUniformityNormalized | logsigma30mm3D_glrlm_RunLengthNonUniformityNormalized |
|  | original_glrlm_RunPercentage | logsigma10mm3D_glrlm_RunPercentage | logsigma30mm3D_glrlm_RunPercentage |
|  | original_glrlm_RunVariance | logsigma10mm3D_glrlm_RunVariance | logsigma30mm3D_glrlm_RunVariance |
|  | original_glrlm_ShortRunEmphasis | logsigma10mm3D_glrlm_ShortRunEmphasis | logsigma30mm3D_glrlm_ShortRunEmphasis |
|  | original_glrlm_ShortRunHighGrayLevelEmphasis | logsigma10mm3D_glrlm_ShortRunHighGrayLevelEmphasis | logsigma30mm3D_glrlm_ShortRunHighGrayLevelEmphasis |
|  | original_glrlm_ShortRunLowGrayLevelEmphasis | logsigma10mm3D_glrlm_ShortRunLowGrayLevelEmphasis | logsigma30mm3D_glrlm_ShortRunLowGrayLevelEmphasis |
|  |  |  |  |
| glszm | original | logsigma10mm3D | logsigma30mm3D |
|  | original_glszm_GrayLevelNonUniformity | logsigma10mm3D_glszm_GrayLevelNonUniformity | logsigma30mm3D_glszm_GrayLevelNonUniformity |
|  | original_glszm_GrayLevelNonUniformityNormalized | logsigma10mm3D_glszm_GrayLevelNonUniformityNormalized | logsigma30mm3D_glszm_GrayLevelNonUniformityNormalized |
|  | original_glszm_GrayLevelVariance | logsigma10mm3D_glszm_GrayLevelVariance | logsigma30mm3D_glszm_GrayLevelVariance |
|  | original_glszm_HighGrayLevelZoneEmphasis | logsigma10mm3D_glszm_HighGrayLevelZoneEmphasis | logsigma30mm3D_glszm_HighGrayLevelZoneEmphasis |
|  | original_glszm_LargeAreaEmphasis | logsigma10mm3D_glszm_LargeAreaEmphasis | logsigma30mm3D_glszm_LargeAreaEmphasis |
|  | original_glszm_LargeAreaHighGrayLevelEmphasis | logsigma10mm3D_glszm_LargeAreaHighGrayLevelEmphasis | logsigma30mm3D_glszm_LargeAreaHighGrayLevelEmphasis |
|  | original_glszm_LargeAreaLowGrayLevelEmphasis | logsigma10mm3D_glszm_LargeAreaLowGrayLevelEmphasis | logsigma30mm3D_glszm_LargeAreaLowGrayLevelEmphasis |
|  | original_glszm_LowGrayLevelZoneEmphasis | logsigma10mm3D_glszm_LowGrayLevelZoneEmphasis | logsigma30mm3D_glszm_LowGrayLevelZoneEmphasis |
|  | original_glszm_SizeZoneNonUniformity | logsigma10mm3D_glszm_SizeZoneNonUniformity | logsigma30mm3D_glszm_SizeZoneNonUniformity |
|  | original_glszm_SizeZoneNonUniformityNormalized | logsigma10mm3D_glszm_SizeZoneNonUniformityNormalized | logsigma30mm3D_glszm_SizeZoneNonUniformityNormalized |
|  | original_glszm_SmallAreaEmphasis | logsigma10mm3D_glszm_SmallAreaEmphasis | logsigma30mm3D_glszm_SmallAreaEmphasis |
|  | original_glszm_SmallAreaHighGrayLevelEmphasis | logsigma10mm3D_glszm_SmallAreaHighGrayLevelEmphasis | logsigma30mm3D_glszm_SmallAreaHighGrayLevelEmphasis |
|  | original_glszm_SmallAreaLowGrayLevelEmphasis | logsigma10mm3D_glszm_SmallAreaLowGrayLevelEmphasis | logsigma30mm3D_glszm_SmallAreaLowGrayLevelEmphasis |
|  | original_glszm_ZoneEntropy | logsigma10mm3D_glszm_ZoneEntropy | logsigma30mm3D_glszm_ZoneEntropy |
|  | original_glszm_ZonePercentage | logsigma10mm3D_glszm_ZonePercentage | logsigma30mm3D_glszm_ZonePercentage |
|  | original_glszm_ZoneVariance | logsigma10mm3D_glszm_ZoneVariance | logsigma30mm3D_glszm_ZoneVariance |
|  |  |  |  |
| ngtdm | original | logsigma10mm3D | logsigma30mm3D |
|  | original_ngtdm_Busyness | logsigma10mm3D_ngtdm_Busyness | logsigma30mm3D_ngtdm_Busyness |
|  | original_ngtdm_Coarseness | logsigma10mm3D_ngtdm_Coarseness | logsigma30mm3D_ngtdm_Coarseness |
|  | original_ngtdm_Complexity | logsigma10mm3D_ngtdm_Complexity | logsigma30mm3D_ngtdm_Complexity |
|  | original_ngtdm_Contrast | logsigma10mm3D_ngtdm_Contrast | logsigma30mm3D_ngtdm_Contrast |
|  | original_ngtdm_Strength | logsigma10mm3D_ngtdm_Strength | logsigma30mm3D_ngtdm_Strength |
